# Temporal perceptual learning distinguishes between empty and filled intervals

**DOI:** 10.1038/s41598-022-13814-w

**Published:** 2022-06-14

**Authors:** Luhe Li, Yuko Yotsumoto, Masamichi J. Hayashi

**Affiliations:** 1 Department of Life Sciences, The University of Tokyo, Tokyo, 153-8902 Japan; 2 Center for Information and Neural Networks (CiNet), Advanced ICT Research Institute, National Institute of Information and Communications Technology, Suita, 565-0871 Japan; 3 Graduate School of Frontier Biosciences, Osaka University, Suita, 565-0871 Japan

**Keywords:** Human behaviour, Perception

## Abstract

Temporal perceptual learning (TPL) refers to improved temporal performance as a result of training with sub-second intervals. Most studies on TPL have focused on empty intervals (i.e. intervals marked by two brief stimuli); however, scholars have suggested that filled intervals (i.e. intervals presented as continuous sensory inputs) might have different underlying mechanisms. Therefore, the current study aimed to test whether empty and filled intervals yield similar TPL performance and whether such learning effects could transfer mutually. To this end, we trained two groups of participants with empty and filled intervals of 200 ms for four days, respectively. We found that the empty-interval group clearly improved their timing performances after training, and such an effect transferred to filled intervals of 200 ms. By contrast, the filled-interval group had neither learning nor transfer effect. Our results further shed light on the distinct mechanisms between empty and filled intervals in time perception while simultaneously replicating the classical findings on TPL involving empty intervals.

## Introduction

We might assume that orchestra members show equal aptitude in producing beats because their musical training included similar components of time perception and production. Surprisingly, percussionists are more accurate than string musicians at temporal reproduction tasks^1^. One major difference in their musical training is whether they have a demarcated or continuous sensory representation of intervals (i.e. empty versus filled intervals). This finding raises a fundamental question: does the effect of temporal perceptual learning (TPL) depend on the stimulus format used for training?

TPL is a phenomenon through which humans reliably improve their ability to discriminate and produce time after undergoing intensive temporal training in the auditory^2–13^, visual^14–16^ and tactile domains^17,18^. Accurate temporal performance in the sub-second range is crucial to multiple functions besides playing music, which include multisensory perception, language abilities and motor planning. TPL studies over the last three decades have featured training involving thousands of trials on a psychophysical task across several days and have focused on sub-second training durations. Typically, researchers have found that the trained interval generates evident improvements in the threshold indicated by the Weber fraction (WF). Such learning effects can transfer flexibly across spatial locations^15^ and auditory pitches^8^ but at a lesser extent to untrained intervals^6,8^.

To date, TPL studies have demonstrated an unbalanced focus on empty intervals as opposed to filled intervals (Table 1). In broad terms, the empty interval is marked by two brief stimuli, whereas the filled interval involves continuous sensory inputs^19,20^. To cite an example in the auditory domain, the gap between two clicks denotes an empty interval, while a continuous tone represents a filled interval. However, several studies have distinguished behavioural performances between empty and filled intervals in multiple temporal tasks. Regarding the subjective perception of duration, human and rat subjects have perceived filled intervals to be longer than empty intervals^21–23^; however, such filled duration illusions were not upheld under certain experimental conditions^24,25^. Another research stream found that filled intervals led to better discrimination performance of durations^19,26^, but contrary results were obtained through different psychophysical tasks^27^.

**Table 1:** Summary of psychophysical research on TPL. It lists the modality of the trained stimuli (A: auditory, T: tactile, V: visual), whether the trained stimulus formats were empty or filled intervals, whether the same trained stimuli produced learning in post-training tests, and whether the learning effect transferred between empty and filled intervals. In reports of successful learning, only one study^15^ used filled intervals. The transfer results had two outcomes: empty intervals transferred to filled intervals (E to F) and not tested (–). To our best knowledge, no study has yet examined the transfer from filled intervals to empty intervals or reported the absence of transfer when tested.

Studies	Modality	Format	Learning	Transfer
Wright et al., 1997	A	Empty	Yes	–
Nagarajan et al., 1998	T	Empty	Yes	–
Westheimer, 1999	V	Filled	Yes	–
Meegan et al., 2000	A	Empty	Yes	–
Karmarkar & Buonomano, 2003	A	Empty	Yes	E to F
Wright & Fitzgerald, 2005	A	Empty	Yes	–
van Wassenhove & Nagarajan, 2007	A	Empty	Yes	–
Grondin et al., 2008	A, V	Empty	No	–
Planetta & Servos, 2008	T	Empty	Yes	–
Lapid et al., 2009	A	Empty	Yes	E to F
Bartolo & Merchant, 2009	A	Empty	Yes	–
Banai et al., 2010	A	Empty	Yes	–
Grondin & Ulrich, 2011	A	Empty	Yes	–
Bratzke et al., 2012	A, V	Empty	Yes	–
Bueti et al., 2012	V	Empty	Yes	–
Chen & Zhou, 2014	A, T, V	Empty	Yes	–
Xu et al., 2021	A	Empty	Yes	–

Despite the disparate effects of the stimulus format on various temporal tasks, it remains unclear whether or not empty and filled intervals show similar performance in TPL, which is a critical task for understanding the neural plasticity of time perception. Moreover, while comparing two interval properties in terms of TPL performance could only suggest differences in learning efficiency, an examination of the transfer effect addresses the critical question of whether empty and filled intervals share a dedicated temporal mechanism.

This study aimed to examine the potential differences between empty and filled intervals in TPL in the visual domain. We focused on two research questions: First, are the learning performances of empty and filled intervals similar? Given the scarce literature on learning via filled intervals, we sought to replicate the paradigm of empty intervals for filled intervals. We hypothesised that although learning may occur for both empty and filled intervals, they could show differences in learning efficiencies (i.e. percentage of improvement). Second, does the learning effect of empty and filled intervals transfer mutually in TPL? This transfer-effect question evaluates whether they share the exact temporal mechanism. Therefore, we included multiple untrained conditions in the pre- and post-tests to examine the transfer effect.

## Results

**Figure 1:**
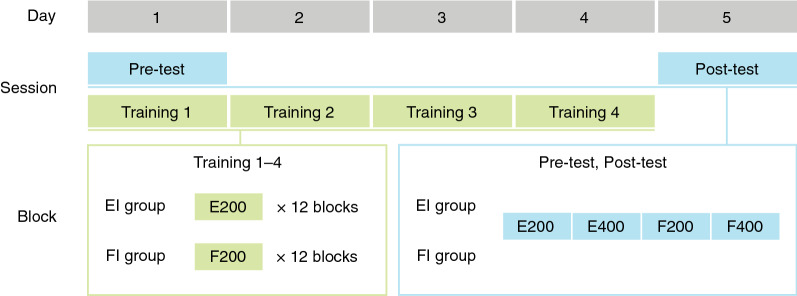
The experimental schedule. The empty-interval (EI) group and the filled-interval (FI) group shared the same pre- and post-tests including four blocks: empty-200 ms (E200), empty-400 ms (E400), filled-200 ms (F200), and filled-400 ms (F400). Across the training sessions, the EI group was trained to discriminate empty-200 ms intervals, and the FI group practiced with filled-200 ms intervals for 12 blocks per session.

Twenty-seven participants underwent temporal perceptual training for four consecutive days and took threshold measurement tests before and after training (Fig. 1 and “Methods”). To investigate the learning effect, we separated them into the empty-interval (EI) and the filled-interval (FI) groups. To assess the transfer effect, both groups received the same threshold tests on two stimulus formats (EI and FI) and two test durations (200 and 400 ms), resulting in four conditions for each group before and after training.

**Figure 2:**
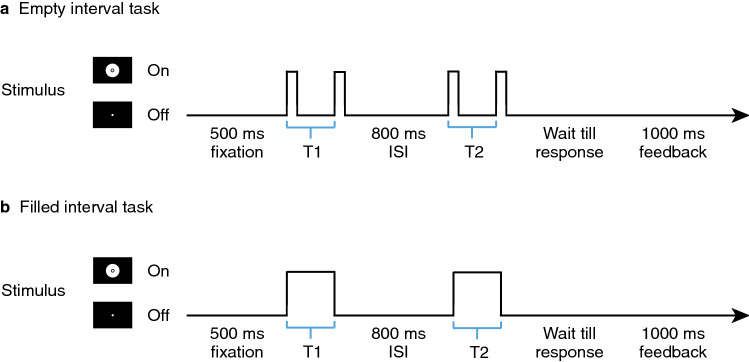
The duration discrimination task for empty (**a**) and filled (**b**) intervals. T1 and T2 represent two sequentially presented intervals: a standard interval (T) and a comparison interval (T + Δt). The presentation order was randomised across trials. We followed the one-up-three-down staircase method, changing Δt according to the responses obtained in previous trials (see “Methods” for details). ISI: inter-stimulus interval.

This study ran a duration discrimination task following a one-up-three-down staircase method in training and tests (Fig. 2 and “Methods”). Throughout the training and test sessions, the EI group was presented with four successive disc flashes, and participants subsequently compared the interval between the first and second discs with that between the third and fourth. Meanwhile, the FI group was presented with two sequentially separated discs, each of which remained on the screen for hundreds of milliseconds, and they compared the two intervals at which the discs were visible. The remaining structure of the task was the same for both groups. The participants received feedback indicated by a colour change in the fixation point after making an unspeeded response.

### Learning effect

**Figure 3:**
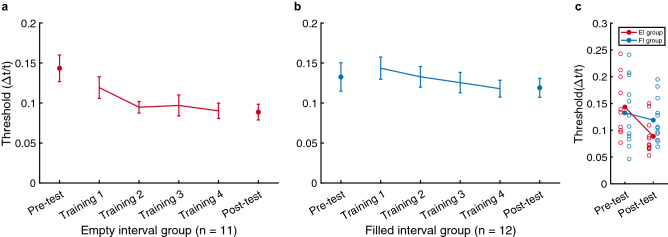
The learning effects of the empty-interval (EI) group (**a**) and the filled-interval (FI) group (**b**). The learning curve shows the change from the pre-test in trained interval thresholds (empty-200 ms for the EI group, filled-200 ms for the FI group) across four training sessions and to the post-test. (**c**) used the same data on the left figures to compare the pre- and post-test mean thresholds of the two groups with individual thresholds presented by scattered points. Error bars indicate between-subject standard errors (s.e.m).

To examine the learning effect in trained interval (i.e. empty-200 ms for the EI group, filled-200 ms for the FI group), we calculated the WF (Δt/t) to obtain thresholds for the pre- and post-tests as well as for each training session (Fig. 3a,b). Supplementary Figs. S1 and S2 report the individual learning curves. We conducted a 2 × 2 repeated-measures analysis of variance (ANOVA) with the two groups (EI group, FI group) as the between-subject variable and sessions (pre-test, post-test) as the within-subject variable (Fig. 3c). We observed a significant main effect of session (F_1,21_ = 10.46, *p* = 0.004, η^2^ = 0.11) but not of groups (F_1,21_ = 0.31, *p* = 0.59), which indicates the effectiveness of temporal perceptual training for both groups. We also found a marginal interaction effect between session and group (F_1,21_ = 3.81, *p* = 0.064, η^2^ = 0.04). Through post-hoc multiple comparisons with Bonferroni corrections, we observed a significant decrease from the pre- to post-test thresholds in the EI group (t_10_ = 4.26, *p* = 0.003) but not in the FI group (t_11_ = 0.82, *p* = 0.86). Our parallel Bayesian paired-samples t-test results showed anecdotal evidence that the FI group threshold did not decrease after training (BF_10_ = 0.59).

### Transfer effect

**Figure 4:**
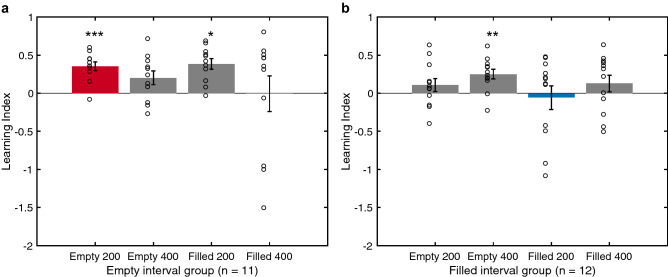
The transfer effects of four conditions for the empty-interval (EI) group (**a**) and the filled-interval (FI) group (**b**). The learning index (LI) is defined as the percentage change in the pre-test threshold (i.e. (pre–post)/pre threshold). A positive LI value indicates improvement after training. The red and blue bars indicate the trained condition for each group whereas the other three grey bars represent the untrained conditions. Dots signify individual learning indices. Error bars specify between-subject standard errors (s.e.m.). The asterisks on each bar denote significant results from one-sided, one-sample t-tests with Bonferroni corrections. *p < .05, **p < 0.01, ***p < 0.001.

To examine the transfer effect, we calculated the learning index (LI) as the percentage change in the pre-test threshold (i.e. (pre–post)/pre threshold) (Fig. 4). A positive LI value suggested an improvement in performance. We then conducted a mixed 2 × 2 × 2 ANOVA with stimulus formats (EI, FI) and test duration (200 ms, 400 ms) as within-subject variables and groups (EI group, FI group) as the between-subject variable. We observed only a main effect of group (F_1,84_ = 4.45, *p* = 0.038, η^2^ = 0.05) without other main effects or interactions. To check whether performance improved after training through the eight conditions as indicated by positive LI, we further conducted one-sided post-hoc one-sample t-tests with Bonferroni corrections. Figure 4a shows that the EI group whose training was on the empty-200 ms interval significantly improved in the empty-200 ms (t_10_ = 5.97, *p* < 0.001) and filled-200 ms conditions (t_10_ = 3.52, *p* = 0.02), suggesting a learning and transfer effect. A Bayesian paired samples t-test found anecdotal evidence that empty-200 and filled-200 conditions evinced the same LI (BF_10_ = 0.43), implying a complete transfer. No significant threshold decrease was observed in the empty-400 ms (*p* = 0.26) or filled-400 ms conditions (*p* = 1).

In the FI group whose training was on the filled-200 ms interval (Fig. 4b), no significant improvement occurred at the filled-200 ms (*p* = 1), empty-200 ms (*p* = 0.92) or filled-400 ms condition (*p* = 1). However, we found a significant improvement in threshold in the empty-400 ms condition (t_11_ = 3.99, *p* = 0.009).

### Learners versus non-learners

The proportion of learners is an important index for task difficulty in perceptual learning. We defined learners as participants whose threshold decreased in the post-test from the pre-test; hence, we found 91% of learners in the EI group in contrast to 67% in the FI group. Our pilot experiment using filled intervals had a learner ratio of 50% with a smaller sample size (Supplementary Fig. S5). The two groups did not differ in their pre-test thresholds (*p* = 0.662, BF_10_ = 0.407); however, a lower learner ratio suggested that the FI task was more difficult to learn than the EI task. Our supplementary analysis of the potential floor effect revealed a significant correlation between the pre-test thresholds (i.e., performance before training) and LI for the FI group but not the EI group, although the correlations between groups were not significantly different (Supplementary Fig. S6).

## Discussion

Using a TPL task in the visual domain, we compared the participants’ performance in discriminating empty intervals and filled intervals. Regarding learning effects, EI training led to improved thresholds whereas FI training demonstrated little to no improvements. Our study was also the first to test the mutual transfer between empty and filled intervals and find an asymmetrical transfer effect. Consistent with the literature, discrimination threshold improvements for empty intervals showed complete transfer to filled intervals, and such transfer was selective to the trained duration. Meanwhile, training with filled intervals did not produce such duration-specific transfer to empty intervals.

A robust learning effect took place using empty intervals, which replicated previous findings. As a classic example, Wright et al.^8^ trained participants using 100 ms auditory stimuli in a duration discrimination task with an adaptive two-interval-forced-choice (2IFC) procedure. They found a clear threshold improvement as indicated by a decrease in the WF from 0.24 to 0.1 (58% improvement). Among the extant studies that have used visual stimuli, our task design was closest to that of Bueti et al.^2^, who used empty-200 ms intervals in a four-day training and found a 38% improvement, a result comparable to our EI outcome of 35%.

Meanwhile, the FI training produced no improvement. Specifically, we trained another group of participants at 200 ms with filled intervals, but a threshold decrease was not observed. To our knowledge, only one study by Westheimer^15^ used filled intervals to investigate TPL. That study used filled visual stimuli of 500 ms in a 2IFC duration discrimination task with constant stimuli methods and reported a 40% threshold improvement. Therefore, we suspect that the small number of reports on FI learning (Table 1) might be due to publication bias in favour of positive results. The discrepancy between our results and Westheimer’s data may be explained by the difference in the intervals used for training (200 versus 500 ms) as different timing mechanisms could be involved for the relatively short and long intervals within the sub-second range^28^. Future studies should clarify this issue by directly comparing the learning efficiencies noted between different sub-second base intervals.

The EI training replicated previous findings that learning effects can transfer to filled intervals with identical or similar durations. Visual perceptual learning studies have employed mean percentage improvement to indicate a complete transfer^29^. We found a complete transfer after EI training marked by a similar LI increase between empty and filled intervals of 200 ms. A similar transfer effect has been reported in the extant TPL studies. For example, Karmarkar and Buonomano^6^ found analogous improvement in two participant groups trained with auditory stimuli of 100 ms and 200 ms empty intervals when tested with filled intervals of corresponding durations. Another study used a staircase duration discrimination task and found that the learning effect of the empty-100 ms interval transferred to the filled-100 ms interval^30^. These results corroborate our finding that the EI learning effect can transfer to filled intervals with the same base duration, suggesting a shared temporal system.

Conversely, we observed no transfer effect from filled intervals to the empty intervals on the trained duration of 200 ms. This result could be ascribed to the insufficient robustness of the learning effect in the first place. Scholars have suggested that learning and transfer are two distinct processes with different time courses^3^. For example, Wright et al.^31^ found that auditory interval training at 100 ms induced significant improvement at the same duration within two days, whereas the transfer to untrained intervals occurred after four days. In other words, there may also be a delay in transfer because learning first occurs to its preferred stimulus features and can only later affect a broader range of neighbouring stimuli after a sufficient time relapse.

Another noteworthy transfer result was the significant improvement in the empty-400 ms interval threshold after training with a filled-200 ms interval. This can hardly be attributed to the learning or transfer effect. Future studies should clarify whether this paradoxical result is robust and determine the mechanisms that could contribute to this effect.

In summary, we suggest two possibilities for this asymmetrical transfer effect. The transfer of learning effect between filled and empty intervals might generally follow a one-way path. Alternatively, non-observation did not dismiss the possibility of mutual transfer. Such transfer may only occur after mature learning, while the lack of learning of filled intervals did not meet this requirement.

The FI group revealed a lower ratio of learners and a stronger floor effect (Supplementary Fig. S6) than the EI group. The two groups did not differ in pre-test thresholds; thus, such discrepancies indicated the higher task difficulty for filled in relation to empty intervals and suggested distinct learning patterns. Specifically, a low threshold before training could restrict learning for filled intervals, whereas the threshold could be improved for empty intervals.

The different learning mechanisms evinced for empty and filled intervals could relate to the temporal dynamics of the visual representation. Empty and filled intervals were reported to evoke different responses in the visual cortex. Empty intervals are bounded by two event onsets that induce two peaks of the event-related field^32^, while filled intervals lead to persistent and stronger activities between the stimulus onset and offset than the baseline in electroencephalography^33^ and magnetoencephalography^32^. Such divergences are further corroborated by a two-temporal-channel model, which proposes different channels for sustained and transient visual stimulations jointly contributing to fMRI responses across visual cortices^34^. These studies could indicate that representations of empty and filled intervals rely on different neural signatures. Our observations of the asymmetrical learning effects and their transfers could thus reflect the differences in the learning difficulty to extract precise time information from transient and sustained responses from visual areas.

Another learning mechanism regarding temporal representation could involve the modulation of the response properties of time-selective neural populations. With regard to the spatial domain, studies have shown sufficient behavioural and neuroscientific evidence that the perceptual learning of low-level visual features such as orientation is mediated by improved selectivity in feature-selective responses in early visual areas^35^. In terms of the temporal domain, several recent neuroimaging studies have demonstrated the existence of time selectivity in the frontoparietal cortices^36–38^. Importantly, scholars have found that inferior parietal cortex activity is correlated with time discrimination accuracy^39^ and bias^40^ in perceived filled intervals. These findings suggest that the parietal region may play a crucial role in timing performance. Future studies should clarify whether empty and filled intervals share time-selective neural populations and whether TPL changes the neural tuning curves of temporal networks through sharpening or amplification.

The sample size is a limitation of the current study because it was difficult to recruit participants during the pandemic. Additionally, it is beyond the scope of this behavioural study to clarify the neural mechanisms of the learning of empty and filled intervals, which calls for neuroimaging approach.

Through a comparison of empty and filled intervals in TPL, we found that the stimulus format led to distinct learning and transfer performances. While empty intervals induced robust learning and could transfer to filled intervals with the same base duration, filled intervals demonstrated neither effect. Such a distinction suggests that empty and filled intervals, despite being different only in the stimulus format, might be learned differently at the temporal stage. These results propose that future studies should rethink the distinction between empty and filled intervals.

## Methods

### Subjects

There were 28 original participants, but one did not show up. Data were collected from the remaining 27 university students (9 male) aged between 18 and 30 with normal or corrected vision. Four participants were further excluded, as they did not pass the data analysis criteria (see Procedures for details). Therefore, data from the remaining 23 participants were used to finalise the results. All participants provided informed written consent before the experiment and received monetary compensation. The institutional review boards of the University of Tokyo approved the study. All experiments were performed in accordance with university guidelines and regulations.

### Apparatus and stimuli

We presented a white disc (4.98 deg diameter) on a CRT display with a 100 Hz refresh rate. For the empty interval, the interval onset and offset were marked by a brief disc flash lasting for two frames (i.e. 20 ms). Specifically, the designated duration for the empty interval was defined as the onset-to-onset interval between two disc flashes. For the filled interval, the disc would remain on the screen for the designated duration. We used an oscilloscope to verify the accurate temporal presentation of stimuli.

The participants conducted the experiment in a soundproof darkroom and were instructed to place their head on a chin rest with a viewing distance of 57 cm and to remain fixated on the central point. We used MATLAB version R2021a and Psychtoolbox-3^41^ to generate all stimuli and collect responses through a conventional keyboard.

### Procedures

Each participant underwent a pre-test (day 1), training (day 1–4), and a post-test (day 5). In the pre- and post-tests, we ran a staircase duration discrimination task with a 2IFC paradigm to estimate the threshold. There were one between-subject variable (EI group, FI group) and three within-subject variables: session (pre-test, post-test), stimulus format (EI, FI), and test duration (200 ms, 400 ms). Accordingly, we obtained the thresholds of these eight conditions for the two groups, with each condition containing one block of 60 trials. Before the pre-test, participants performed 10 practice trials to familiarise themselves with the task.

For the training, the participants were grouped into two, practicing on empty and filled intervals at 200 ms, respectively. They rehearsed the same duration discrimination task except that the presentation of the trained interval was demarcated or continuous. The EI group compared the inter-flash intervals between the first and second discs and between the third and fourth. Meanwhile, the FI group compared two intervals at which the disc stayed on the screen. There were 12 blocks of 60 trials, yielding 720 trials per day. In total, the participants completed 2,880 trials across the four-day training.

The other task design was the same for the EI and FI groups. The design of the duration discrimination task was inspired by Bueti et al.^2^. The fixation point (0.4 deg diameter) stayed at the centre of the screen throughout the experiment. In each trial, after a short fixation period (500 ms), two interval stimuli were sequentially presented, separated by a fixation-only inter-stimulus interval (ISI) (800 ms). These two intervals were a standard interval (T) and a comparison interval (T + Δt) presented with a random sequence. After the visual stimulus for the second interval was extinguished, the participant made an unspeeded response regarding which interval was longer. A change in the colour of the fixation point immediately provided feedback to the response: the fixation point turned green if the response was correct, otherwise red. The feedback lasted for 1000 ms followed by the subsequent trial.

The duration discrimination task was coupled with an adaptive staircase method. To obtain the Δt threshold at 79% accuracy, we adjusted the comparison interval (T + Δt) trial-wise by following the one-up-three-down procedure^42^. If the participants made three consecutive correct responses, the comparison interval would decrease; if they made one incorrect response, the comparison interval would increase. The initial Δt was 20% of the standard interval (e.g. 40 ms if the standard interval was 200 ms) and was changed in steps. The initial step size of the Δt change was 10% of the standard interval. After the third reversal, the step size was updated to 5% of the standard interval to approximate the threshold more precisely.

### Data analysis

We calculated the threshold of each block by averaging the reversal point values excluding the first three reversals. If there were fewer than five reversals, the data of the whole block would be excluded. No block was excluded following this criterion. We then divided the threshold by the respective standard interval (T) to obtain the WF as the standard measurement of the temporal threshold. In the pre- and post-tests, thresholds were estimated for four conditions for the two groups. In the training, we obtained thresholds of 12 blocks for 4 sessions and excluded a block if its value was an outlier within each session (i.e. more than three scaled median absolute deviations away from the group mean). We averaged the remaining thresholds across blocks to obtain the threshold for each training session.

We also excluded participants if their thresholds in more than one session were three standard deviations larger than the group means. As a result, each group had two participants excluded, resulting in 11 participants in the EI group and 12 participants in the FI group. We also conducted the same analysis with no participants excluded, resulting in the same findings as in the main analysis (Supplementary Figs. S3, S4). Based on the thresholds across pre- and post-tests and training sessions, we then plotted the individual learning curves (Supplementary Figs. S1, S2) and the group average learning curves (Fig. 3) for each group. We used MATLAB version R2021a for statistical analysis and conducted parallel Bayesian tests using JASP version 0.15 when necessary.

## Supplementary Information

Supplementary Information.

## Data Availability

The data and analysis scripts are available at https://osf.io/3bm9a/.
