# Supplementary material for: Temporal perceptual learning distinguishes between empty and filled intervals
Source: Sci Rep. 2022 Jun 14;12:9824. doi: 10.1038/s41598-022-13814-w (PMC9198236; doi:10.1038/s41598-022-13814-w)
Supplement: Supplementary file 1 — Supplementary Information. [file 41598_2022_13814_MOESM1_ESM.docx]

Supplemental Materials: Temporal perceptual learning distinguishes between empty and filled intervals

Luhe Li^1,2^, Yuko Yotsumoto^1^, Masamichi J. Hayashi^2,3^*

^1^Department of Life Sciences, The University of Tokyo, Tokyo 153-8902, Japan

^2^Center for Information and Neural Networks (CiNet), Advanced ICT Research Institute, National Institute of Information and Communications Technology, Suita 565-0871, Japan

^3^Graduate School of Frontier Biosciences, Osaka University, Suita 565-0871, Japan

*Corresponding author

**Figure S1.** The individual learning curve of the empty-interval group (n = 11).

**Figure S2.** The individual learning curve of the filled-interval group (n = 12).

**Figure S3.** Results without participants exclusion: learning effect of the empty-interval group (EI; a; n = 13) and the filled-interval group (FI; b; n = 14). In the main analysis, we excluded participants if their threshold in more than one session was larger than three standard deviations than the group means. Consequently, there were two excluded participants in each group. Here, we performed the same analysis with all participants. Same as Figure 2, the learning curve shows the change of thresholds of trained intervals interval (i.e., empty 200 ms for EI group and filled 200 ms for FI group) from pre-test, across four training sessions, to post-test. Error bars indicate between-subject standard error (s.e.m).

**Figure S4.** Results without participants exclusion: transfer effect of four conditions of the empty-interval group (a; n = 13) and the filled-interval group (b; n = 14). In the main analysis, there were two excluded participants in each group. In this instance, we performed the same analysis with all participants. Learning index is defined as the percentage change of pre-test threshold, which is post-threshold subtracted from pre-threshold and divided by pre-threshold. A positive value indicates improvement after training. The coloured bar indicates the condition of trained interval in each group, whereas the other three grey bars are untrained interval conditions. Error bars indicate between-subject standard error (s.e.m).

**Figure S5.** Learning curves in the pilot experiment using only filled intervals (n = 6): (a) individual learning curves and (b) group average learning curves. The experimental design was the same as the main experiment except for the following. Two groups of participants underwent the temporal training task on filled-442 ms (solid line) and filled-650 ms (dashed line) intervals, respectively. They practiced 10 blocks of 60 trials for 4 consecutive days in the training. The task was the same as the training in pre- and post-tests except that no feedback was provided. The inter-stimulus interval was jittered between 400 to 600 ms. There was no significant improvement in the two groups. Learners were defined as those who had a lower threshold in the post-test than pre-test. There were two learners in the filled-442 ms training group and one learner in the filled-650 ms training group, resulting in a total learner ratio of 50%. Error bars indicate between-subject standard error (s.e.m).

**Figure S6.** Spearman’s correlation between pre-test thresholds and learning index (LI) for the empty-interval (EI) group (a) and the filled-interval (FI) group (b). Figures S6a and S6b plot the individual LI against pre-test thresholds (empty-200 ms for the EI group, filled-200 ms for the FI group) and the least-square fit line. Figure S6c shows the difference distribution (EI - FI) of correlation coefficients bootstrapped for 10,000 trials. Solid lines indicate 95% confidence intervals. The dashed line indicates the group difference of Spearman’s correlation. We found a significant correlation between pre-test thresholds and LI in the FI group (r_s_ = 0.762, p = 0.004, 95% CI = 0.29–0.95; Figure S6b), but not in the EI group (r_s_ = 0.460, p = 0.154, 95% CI = −0.26–0.92; Figure S6a). To compare independent correlations between groups, we also computed the difference distribution (EI - FI) between bootstrapped correlations. The difference CI contained 0 and did not reach statistical significance at the 5% level (95% CI = −1.03–0.37, p = 0.398; Figure S6c), suggesting that the correlations were not qualitatively different between EI and FI groups. Together with the significant correlation in the FI group, we conclude that the pre-test threshold may predict the degree of learning, at least in the FI group. **p < 0.01.
